# Distinguishing false and true positive detections of high frequency oscillations

**DOI:** 10.1088/1741-2552/abb89b

**Published:** 2020-10-09

**Authors:** Stephen V Gliske, Zihan Qin, Katy Lau, Catalina Alvarado-Rojas, Pariya Salami, Rina Zelmann, William C Stacey

**Affiliations:** 1 Department of Neurosurgery, University of Nebraska Medical Center, Omaha, Nebraska, United States of America; 2 Department of Neurology, University of Michigan, Ann Arbor, Michigan, United States of America; 3 Department of Biomedical Engineering, University of Michigan, Ann Arbor, Michigan, United States of America; 4 Department of Electrical Engineering and Computer Science, University of Michigan, Ann Arbor, Michigan, United States of America; 5 Department of Electronic Engineering, Pontificia Universidad Javeriana, Bogotá, Colombia; 6 Department of Neurology, Massachusetts General Hospital and Harvard Medical School, Boston, Massachusetts, United States of America

**Keywords:** ripple, fast ripple, HFO

## Abstract

**Objective.:**

High frequency oscillations (HFOs) are a promising biomarker of tissue that instigates seizures. However, ambiguous data and random background fluctuations can cause any HFO detector (human or automated) to falsely label non-HFO data as an HFO (a false positive detection). The objective of this paper was to identify quantitative features of HFOs that distinguish between true and false positive detections.

**Approach.:**

Feature selection was performed using background data in multi-day, interictal intracranial recordings from ten patients. We selected the feature most similar between randomly selected segments of background data and HFOs detected in surrogate background data (false positive detections by construction). We then compared these results with fuzzy clustering of detected HFOs in clinical data to verify the feature’s applicability. We validated the feature is sensitive to false versus true positive HFO detections by using an independent data set (six subjects) scored for HFOs by three human reviewers. Lastly, we compared the effect of redacting putative false positive HFO detections on the distribution of HFOs across channels and their association with seizure onset zone (SOZ) and resected volume (RV).

**Main results.:**

Of the 15 analyzed features, the analysis selected only skewness of the curvature (skewCurve). The feature was validated in human scored data to be associated with distinguishing true and false positive HFO detections. Automated HFO detections with higher skewCurve were more focal based on entropy measures and had increased localization to both the SOZ and RV.

**Significance.:**

We identified a quantitative feature of HFOs which helps distinguish between true and false positive detections. Redacting putative false positive HFO detections improves the specificity of HFOs as a biomarker of epileptic tissue.

## 1. Introduction

Over 50 million people worldwide have epilepsy, with about one third of them being unable to obtain seizure control from medications (Kwan and Brodie 2000). For these patients with refractory epilepsy, resective surgery is a primary treatment option. This surgery seeks to remove the region of the brain instigating seizures, the hypothesized epileptogenic zone. As the true epileptogenic zone is unknown, clinicians use multiple modalities to estimate the location of where the seizures start, denoted the seizure onset zone (SOZ). One of the main modalities for identifying SOZ is intracranial EEG, which involves placement of electrodes on the cortical surface or deep within the brain followed by days of hospitalization to observe spontaneous seizures. Unfortunately, less than 60% of patients have a seizure-free outcome after resective surgery informed by intracranial EEG (Edelvik *et al* 2013, Noe *et al* 2013, Yu *et al* 2014).

High frequency oscillations (HFOs), defined as discrete, spontaneous EEG transients with frequency between 80 and 500 Hz, have become a promising new biomarker for identifying the epileptogenic zone, e.g. (Bragin *et al* 2002, Blanco *et al* 2011, Zijlmans *et al* 2011). Removal of interictal HFO-generating areas has been shown to be correlated with the surgical outcome in patients with refractory epilepsy (Akiyama *et al* 2011, Gliske *et al* 2016a, Jacobs *et al* 2010, Haegelen *et al* 2013, Wu *et al* 2010). HFOs can be detected by human scoring or by automated methods, though manual review is extremely time consuming (taking about 1 h to review 10 min on one channel (Zelmann *et al* 2009)) and is inevitably subjective (Gardner *et al* 2007). Additionally, the HFO rate has variability over time (Gliske *et al* 2018), requiring analysis of HFOs over prolonged EEG recordings in order to obtain the most complete information.

While HFOs are a promising biomarker of tissue instigating seizures, all HFO detectors (human and automated) suffer from several common weaknesses: (1) both noise (i.e. signals not due to brain activity) as well as random fluctuations of the physiological brain activity can be falsely marked as HFOs, i.e. false positive detections (Bénar *et al* 2010), (2) not every HFO will be detected (false negative detections), and (3) healthy brain tissue can also produce HFOs that represent normal neural oscillations (Engel *et al* 2009, Khadjevand *et al* 2017). The presence of false positive HFO detections (weakness 1) already implies that not every putative HFO necessarily carries the same weight of information about the epileptogenic zone, which is further confounded by weakness 3, that some HFOs are due to normal brain processes rather than being specific to epilepsy. Mitigating these weaknesses has the potential to improve the clinical utility of HFOs to identify seizure networks. Note that weakness 3 is not referring to false positive HFO detections, but true HFOs that are not due to epileptic processes. The ability to distinguish ‘normal’ from ‘epileptic’ (pathological) HFOs is an ongoing area of research, e.g. Alkawadri *et al* (2014), Cimbalnik *et al* (2018), Khadjevand *et al* (2017) and Liu *et al* (2018).

Weaknesses 1 and 2, the presence of false positive and false negative detections, are inherent to any detection process. HFO detection relies upon finding oscillations that stand out from the background. Non-neural artifacts can often produce fast transients that generate such large oscillations, but with care these artifacts can be ignored by the detector (Gliske *et al* 2016a, Ren *et al* 2019). However, another potential source of false positive detections is a patient’s neural background EEG. The neural background may have high frequency oscillatory activity due to the rich repertoire of underlying physiological activity. In such cases, by sheer statistics there will be certain short periods that emerge from this background, i.e. the upper tail of the distribution of normal brain activity. Both automated methods and human scoring must decide whether such ambiguous data represent a true HFO or just a random fluctuation of the background brain activity. Such choices invariably lead to some false positive detections (background activity falsely marked as an HFO) and false negative detections (an HFO falsely marked as background activity). The focus of this paper relates to false positive HFO detections, not false negative HFO detections.

Identifying these false positive HFO detections due to neural sources is challenging, as there is no gold standard to label a given HFO as a false or true positive. One option is to concentrate on how similar the detected events are to the background itself: false positive detections due to neural sources should have some similar characteristics as the background neural activity. We thus sought to identify quantitative features of the background that were similar to HFOs to disambiguate false and true positive HFO detections.

We identified three approaches for identifying such HFO features, which we combined to overcome the weaknesses inherent in each approach. The first approach was to compute quantitative features of randomly-selected segments of the high-frequency background EEG. This method accurately interrogates the distribution of background features; however, these distributions may not exactly match those of false positive HFO detections because the false positive detections are extreme cases and above a detection threshold. A second approach was to create surrogate background data (i.e. ‘artificial EEG’ based on the actual background) and detect HFOs in these data. By construction, any detected HFO would be a false positive, but we recognized that the surrogate background data generation process may influence the feature distribution. A third approach was to apply a fuzzy clustering method to the putative HFO detections in the clinical data, although the method alone could not identify which, if any, cluster corresponded to false positive detections. We combined these approaches by seeking to identify quantitative features that have similar distributions in the randomly-selected background segments, the HFO detections in surrogate background data, and in one fuzzy cluster of putative HFO detections in clinical data. The existence of such features was not guaranteed. Yet, any features found with similar distributions for all three cases could represent a signature of false positive HFO detections. Specifically, such features would, by construction, identify a subset of HFOs that are similar to (1) randomly-selected segments of background EEG, (2) constructed false positive HFO detections, and (3) a natural, unsupervised cluster of detected putative HFOs.

After finding a quantitative feature with the above properties, we validated whether it truly distinguishes false versus true positive HFO detections and whether redaction of these putative false positive HFO detections improves the utility of HFOs. First, we used the information that while human reviewers do mark some false positive HFO detections, events with greater consensus among reviewers are more likely to be true positive HFOs. Thus, we compared the distribution of the identified quantitative feature for events marked by only one of three human reviewers with events marked by at least two of the three human reviewers. Second, the association of HFOs with SOZ and resected volume (RV) in good surgery outcome patients is expected to increase after removal of false positive HFO detections. We thus removed the events predicted by the quantitative feature to be false positive HFO detections and assessed how this changed the association of HFO rate with SOZ and RV.

## 2. Methods

### 2.1. Patient selection

Subjects were selected from patients with medically refractory epilepsy at the University of Michigan who underwent intracranial EEG monitoring in preparation for epilepsy surgery. Two cohorts of subjects were identified. Cohort 1 was used for automated detectors using all recorded, valid, interictal data. Cohort 2 was used for manual scoring and was selected to be an independent set of subjects. In both cohorts, we selected subjects with clinical data acquisition at > 4 kHz sampling rate. Selected subjects all had data acquired on a Natus Quantum (Natus Medical Inc) with a sampling rate of 4096 Hz and 1200 Hz anti-aliasing filter. For Cohort 1 (automated processing), we selected subjects meeting the following criteria as of April, 2019: International League Against Epilepsy (ILAE) Class I surgery outcome after at least one year post-resection. Subjects which were Class I when compliant with medications were considered Class I for all analyses in this paper. This yielded 11 subjects, one of whom later changed to ILAE Class II and whose data were subsequently removed from Cohort 1. Thus, ten subjects (six of whom were adults) were included in Cohort 1. In the Cohort 2 (manual scoring), we selected the first six consecutive patients (five of whom were adults) which did not have an ILAE Class I surgery outcome. Demographic and subject characteristics are shown in Table 1.

All data were acquired with approval of the local Institutional Review Board (IRB) and consent/assent was obtained from all subjects and guardians for the deidentified data to be analyzed for research use. For each patient, the clinically determined SOZ was determined from the final clinical report, written by the treating clinicians. The RV and surgery outcome were determined by discussion with the neurosurgeon performing the resection and the clinical metadata. Only data further than 30 min from the start of a clinical seizure were included in the analysis and results, with seizure times obtained from the clinical reports. Data with ambiguities in seizure times were ignored. Subsets of the raw data were reviewed by a board certified epileptologist, and channels with obvious poor quality or known to be extraparenchymal were excluded from the analysis. Additionally, data were excluded during and near times coinciding with testing wire connections and conducting mapping procedures.

### 2.2. Preprocessing

#### 2.2.1. General preprocessing

**Figure 1.**
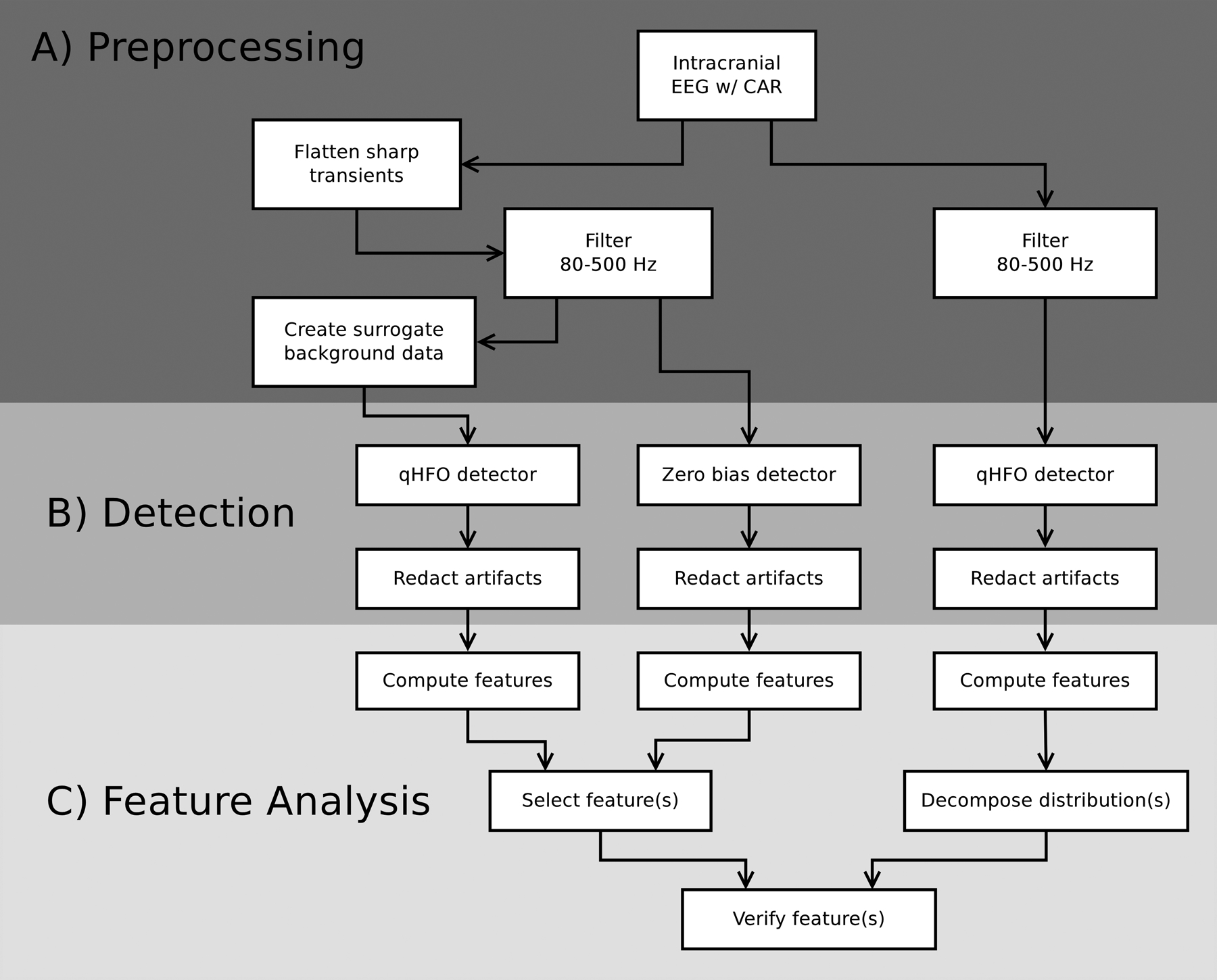
Analysis flow chart for Cohort 1. The analysis is divided into preprocessing (A), detection (B), and feature analysis (C) stages. The preprocessing and detection stages of the farthest right data stream represents the HFO processing used our previous publications. The left two data streams are based on analysis of the EEG background data agnostic to the HFO detections in the clinical data, the farthest left being HFOs detected in surrogate background data and the middle column being zero bias detections (random samples of the background data). Abbreviations: CAR, common average reference.

All data were preprocessed by re-referencing and filtering. See figure 1(A). The common average reference was selected in previous work to reduce noise (Gliske *et al* 2016a), and separate references were used for depth and ECoG channels. To select high frequency data, we used a 10th order, bidirectional elliptical passband filter, 80–500 Hz passband, 0.5 dB passband ripple, and 65 dB stopband ripple. These steps are the same as in our previous HFO work (Gliske *et al* 2018, 2016a, Ren *et al* 2019).

#### 2.2.2. Flattening of sharp transients

An additional preprocessing step was needed for the surrogate data generation, as there exist small sharp transients in the data that appear as instantaneous shifts in the DC offset. These shifts are small enough not to cause false positive HFO detections, and thus they are below threshold to be identified by the artifact detection algorithms (Gliske *et al* 2016a). However, these transients negatively impact the surrogate data generation, which is based on directly modeling the background data (see section 2.2.3). We thus ‘flattened’ the data and minimized the effect of these transients for surrogate data generation by computing the mean and standard deviation of the difference of adjacent samples in the signal on each channel in each 10-minute epoch. Any pair-wise difference with magnitude greater than 10 standard deviations from the mean had its magnitude scaled to be exactly 10-standard deviations from the mean, resulting in a DC shift of the following data to counter the transient. This step was applied after the common average reference and before the bandpass filter for surrogate background generation (see section 2.2.3) and for zero bias events (see section 2.3.3), to allow a direct comparison to the HFOs detected in the surrogate data. We verified the step had minimal impact on the feature distribution of HFOs detected in the actual EEG data (results not shown). Thus, to be consistent with previous work (e.g. Gliske *et al* 2016a, Ren *et al* 2019, Gliske *et al* 2018), the flattening step was not applied before processing HFOs in the actual EEG data. See figure 1(A).

#### 2.2.3. Generation of surrogate background data

**Figure 2.**
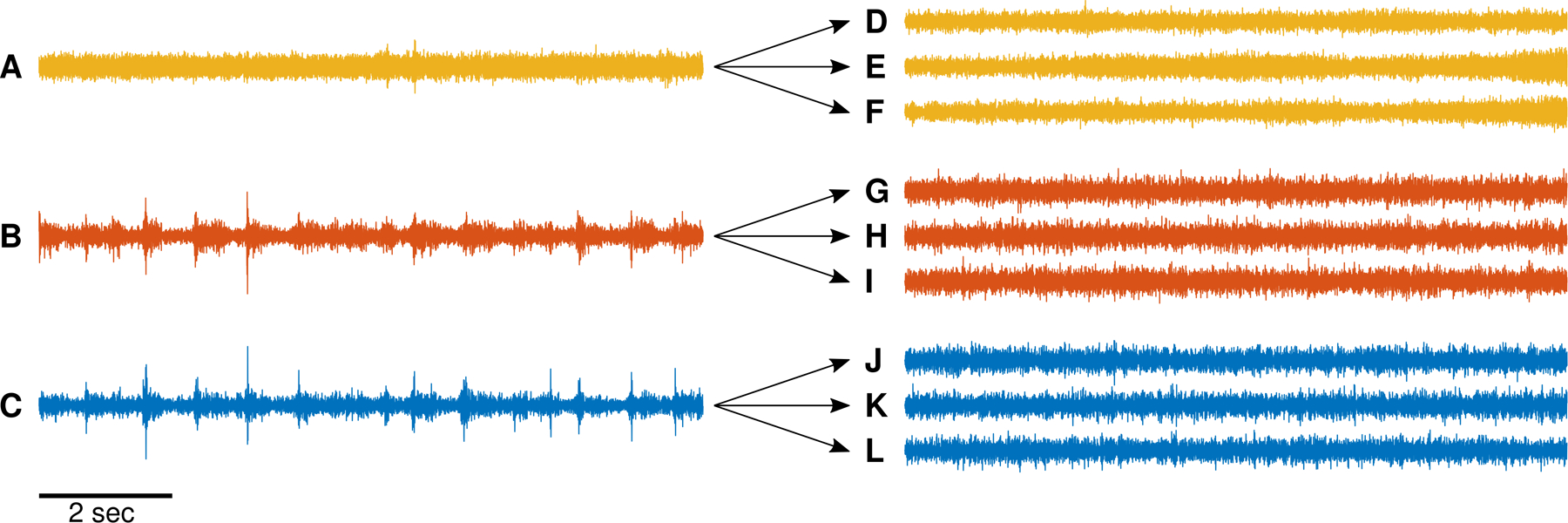
Example surrogate data. Ten seconds of intracranial EEG data from three depths electrodes were filtered in the 80–500 Hz range using the same filter settings as for qHFO detection (A)-(C). From each of these 10 s epochs of data, we generated three surrogates of the background (D)-(L). Note the sharp, transient high amplitude events in panel B and C (putative HFOs) are not present in panels (G)-(L). The vertical scale is arbitrary, but consistent for all panels.

Surrogate background data were created in order to construct HFO detections that are known to be false positives. The surrogate data was created by using linear autoregressive models, as has been done with intracranial EEG data in other publications, e.g. (Roehri *et al* 2017). This procedure utilized a mathematical similarity between the linear autoregression models and an all-pole digital filter. Specifically, fitting a linear autoregressive model to a specific set of data results in a set of coefficients and a noise amplitude. These coefficients are used to filter white noise with the same noise amplitude, resulting in surrogate data with the same properties (autoregressive coefficients) as the original data. See example data in figure 2.

We generated surrogate data as follows. We focused on 10-minute epochs of data at a time, consistent with the HFO detection algorithm (section 2.3.1). Autoregressive coefficients (order 200) were estimated for each 10 s section of data (40 960 samples) using the Burg Algorithm (Burg 1968). The Burg method is based using forward and backward least squares estimates for the autoregression coefficients. These coefficients were then used to make 20 copies of surrogate data. To avoid discontinuities in the data, 10.1 s of surrogate data were produced per each 10 s of clinical data for each copy, allowing a 0.1 s overlap between consecutive sections of surrogate data. During the overlap, the signals were multiplied by linear functions, one scaling from 1 to 0 and the other from 0 to 1, resulting in a smooth transition. Once a full 10 min of surrogate data were generated on all channels, the full qHFO detection algorithm was applied (see section 2.3.1).

Note, computational requirements for this process were quite high, even with highly efficient C++ code utilizing parallelization. A typical subject with 50 channels took about 15 min of wall time to compute 20 surrogate copies of 10 min of actual EEG data when using 10 cores on the Great Lakes high performance computing cluster at the University of Michigan. Thus, while one surrogate could be generated and processed in less than 1 cpu-minute per 10 min of recorded data, the 20 surrogates for one week of 50 channels of data (a typical amount for one patient) corresponds to over 2 cpu-years of processing. The processing of the ten subjects analyzed in this paper required multiple cpu-decades of processing.

### 2.3. Detection algorithms

The analysis utilized multiple types of detection algorithms. Detectors include automated and manual HFO detectors and a ‘detector’ which randomly selected samples of the background data, denoted the zero bias detector. The automated detectors were applied to Cohort 1 data (see figure 1 (B)) and both automated and manual scoring of HFOs were applied to Cohort 2 data.

#### 2.3.1. Automated HFO detector

HFO detections were found using the previously published and validated qHFO detector (Gliske *et al* 2016a). After preprocessing (common average reference and bandpass filter), it then applies the Staba HFO detector (Staba *et al* 2002) to create a putative set of HFO detections. As the Staba HFO detector is based on 10 minute windows of data, all automated detectors were set to process 10-minutes of data at a time. The qHFO method additionally identifies sharp-transient artifacts and HFO detections on the common average itself and redacts any putative HFO detections concurrent with those artifacts to yield the final set of ‘quality’ HFO detections. Thus, the qHFO detector already removes false positives due to sharp transient artifacts and filtering effects.

#### 2.3.2. Manual scoring of HFOs

Three experts in manual scoring of HFOs (C.A-R., P.S., R.Z.) identified HFOs completely blind to all of the other analysis described in this paper. Experts were given two channels of interictal data per subject, 20-minute duration, for each subject in Cohort 2, along with data for other subjects who did not meet inclusion criteria for Cohort 2 (used in other studies). The same common average reference used for automated detections was applied before extracting the two channels, but no other filters were applied when the data was prepared. Experts were instructed to detect HFOs according to their own best practice.

#### 2.3.3. Zero bias detector

Zero bias detectors randomly identify segments of the background data and are utilized in other fields such as high energy physics, e.g. (Adamczyk *et al* 2012). Zero bias detectors are agnostic to the actual data (i.e. whether they select an event at a given time is not biased by the data at that time), and thus events are not technically ‘detected’ but are selected at random. We implemented a zero bias detector by randomly selecting the time for 10 detections per each 10-minute epoch of each channel. We set each zero bias detection to have 30 msec duration, corresponding to a typical HFO duration. In the analyzed set of qHFO detections in the clinical data, the median HFO duration was 30.0 msec, with the 25th percentile being at 23.4 msec and the 75th percentile being at 41.0 msec. Zero bias events concurrent with artifacts were redacted, but not those concurrent with HFO detections. It is possible that some zero bias detections may have overlapped with HFO detections, but this was expected to occur less than once per 1000 zero bias events (Monte Carlo simulation based on assuming 30 msec mean HFO duration and an abnormally high rate of 1 HFO min^−1^).

### 2.4. Feature analysis

The goal of feature analysis was to use all the data prepared in the earlier analysis stages to identify a quantitative feature that is sensitive to whether an HFO detection is a true or false positive. For the relationship between analysis stages, see figure 1. Feature analysis involved two parts. First, we selected features based on the zero bias and surrogate-data HFOs. Second, we verified that the feature distributions of those events correspond to a natural cluster of the HFO detections in the clinical data. Note, features are selected in an unsupervised fashion based on the feature having similar distributions for difference event types (as explained in section 2.4.2). No explicit supervised training was used at any time in this analysis.

#### 2.4.1. Quantitative feature computation

Features were computed for all detections using the 80–500 Hz bandpass filtered data sets. For the feature selection step, a total of 15 features were computed and assessed. Other feature analysis steps included just the subset of the 15 features that were selected. The 15 features include the power (standard deviation of the signal), absolute value of the skewness, and the kurtosis of the time-domain signal (three features). We also computed the mean, standard deviation, skewness and kurtosis of three transforms of the data: absolute value of the difference of consecutive samples (related to the slope, first derivative), absolute value of the difference of the difference of consecutive samples (related to the curvature, second derivative), and the Teager-Kaiser Energy Operator (the sample value squared minus the product of the adjacent samples). Note, for all features except power, the signal was normalized to have unit power before computing the features.

#### 2.4.2. Feature selection

We sought to identify features with similar distributions in two of the three data streams, specifically, in both random samples of the background EEG and the constructed false positive HFO detections. While there are many definitions of ‘similarity’ between probability distributions, the definition we selected was the extent to which one could accurately classify events drawn from a mixture of the two distributions. There are a number of options available to quantify this similarity without actually using a specific learning algorithm. If we were working in higher dimensions, we could have used the Henze-Penrose statistic, related to the Henze-Penrose divergence (Moon *et al* 2015, Berisha *et al* 2016, Gliske *et al* 2016b). As we are in one dimension for each comparison, area under the receiver operator curve (AUROC) provides a simpler and still directly interpretable means of quantifying the difference between two distributions (Hanley and Mcneil 1982, Brown and Davis 2006, Kallner 2018). AUROC values of 0.5 correspond to complete similarity between the distributions, whereas values of 0.0 or 1.0 represent completely disjoint distributions. For each of the 15 quantitative features (section 2.4.1), the AUROC between the distribution of zero bias events and the distribution for HFOs detected in the surrogate data was computed using the MATLAB *perfcurve* function, directly quantifying the similarity of the two distributions. Since we were not concerned which group (zero bias events or HFOs in surrogate background data) had higher mean, after computing the median AUROC per feature over all patients in Cohort 1, we then corrected any values that were less than 0.5 by taking one minus those values (effectively swapping the order of the two groups).

After quantifying the similarity of the feature distribution for each feature, we then used these AUROC values to select features which were extremely similar between the two event types. We choose to define ‘extremely similar’ as AUROC < 0.55, recognizing that, *a priori*, there is no assurance that any feature would meet this criterion. We also computed the interpatient variability in features of zero bias events, as well as for HFO detections in surrogate background data. In both cases, the median AUROC was 0.54. Thus our threshold for ‘extremely similar’ between the two types of events is just above the level of interpatient variability within each type of event. We also verified that adjusting the parameter within the range 0.53 to 0.60 would have had no effect on the results. Noting that only two features were identified and that they were highly correlated (Pearson correlation coefficient > 0.9), we then selected the feature with AUROC closest to 0.5.

#### 2.4.3. Fuzzy clustering

**Figure 3.**
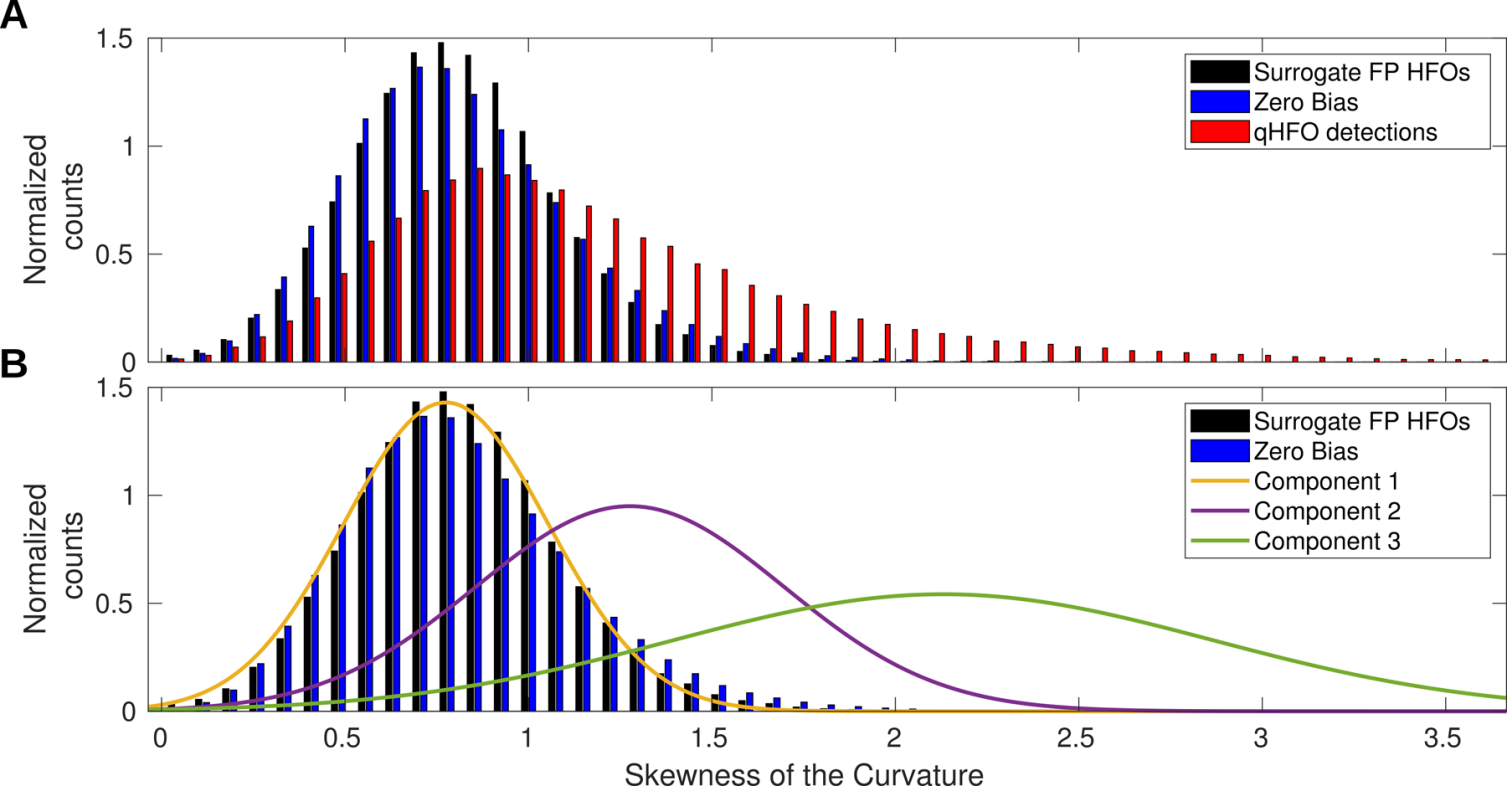
Distribution of skewness of the curvature (skewCurve). The distributions are shown for the HFOs from the surrogate data (black), zero bias events (blue) and for actual HFO detections (red) (A). Panel B repeats showing two of the distributions from panel A for reference, but the distribution for actual HFO events has been replaced by the three normalized components from the Gaussian Mixture Model trained on those events. Component 1 closely matches the distribution for HFOs from surrogate data and for zero bias events, supporting that the qHFO detections in the *clinical data* include a subset of events corresponding to the modeled false positive events (the zero bias and HFO detections in surrogate data).

The next step was to apply fuzzy clustering, in preparation for verifying whether the distribution of the selected feature corresponds with any natural clusters of the HFO detections in the actual intracranial, interictal EEG data. To accomplish this, we used Gaussian Mixture Models, which decomposed the distribution of the selected feature for each patient into multiple clusters; see figure 3. Note this use of a Gaussian Mixture Model is different than the most familiar use of this tool. Instead of using it tool to cluster data, we are using it to decompose a multimodal distribution into individual components. Mathematically these uses are equivalent, despite the conceptual differences. We set the model to have three components (i.e. clusters): one to represent the distribution of putative false positive HFO detections (expected to be approximately Gaussian based on the zero bias analysis) and two others to account for the non-Gaussian distribution of putative true positive HFO detections. We also set the mixture model algorithm to use ten replicates, to avoid effects due to initial conditions, and set the maximum iteration level to 1000 to avoid premature stopping.

#### 2.4.4. Feature verification

The last step of the feature analysis was to verify that HFOs detected in clinical data include a subset corresponding to our model of false positive HFO detections. In general terms, our approach was to (1) select the cluster with distribution most similar to the distribution of background and (2) quantify exactly how close it was (as none may be very close). Explicitly, we first identified one of the fuzzy clusters as the ‘false positive cluster’ by selecting the cluster with mean closest to the mean of the distribution for zero bias events. Even though we selected the cluster with the mean closest to the mean of the background data, there is no guarantee that the distribution of that cluster is similar to the distribution of the background data. Thus, we then compared this cluster with the distributions of zero bias events and the HFO detections in the surrogate background data. The comparison was quantified using AUROC (Matlab *perfcurve* function, with weights for actual HFOs set to the posterior probability of being in the ‘false positive cluster’).

### 2.5. Validation

Having identified a feature and verified its correspondence with a natural subset of HFO detections in the clinical data, the next step was to validate that the subset was actually associated with false positive HFO detections. We do this by analyzing HFO detections scored by three human reviewers. We also validated that removing putative false positive HFO detections improve specificity of HFOs to the SOZ and RV.

#### 2.5.1. Human-scored HFOs

Although human reviewers are also susceptible to false positive detections (marking data as an HFO when it is not), events with greater consensus among reviewers are more likely to be true positive HFO detections. Thus, while human markings are not an ideal gold standard for training how to recognize false positive detections, they still serve as an excellent metric to validate that the feature selected through other means is associated with false positive HFO detections. We specifically assessed whether the selected feature distinguishes between events with consensus (marked by at least two of the three reviewers) with events without consensus (marked by only one reviewer). Additionally, we used the human scored HFOs in Cohort 2 as an independent training data to select a threshold for identifying false positive HFO detections. We then assessed the effect of this threshold in the independent and larger Cohort 1.

Data processing was as follows. The selected feature was computed for each human marked HFO in Cohort 2, as well as for the automated HFO detections within the exact data given to the human reviewers. We additionally formed a set of consensus and non-consensus events and computed the selected feature for each of these events. One cannot simply split the events into those marked by one reviewer and those marked by more than one reviewer, as the reviewers are viewing continuous data, not discrete events. For example, one reviewer could mark a section of data as an HFO, while another reviewer could mark two subsets of that period as HFOs. Thus, to identify the consensus versus non-consensus events, we first created the union of all times marked as HFOs by any of the three reviewers. Events in this union which overlap with HFO markings of only one reviewer were labeled non-consensus events, with the remainder labeled as consensus events. We then noted the median feature value for consensus and non-consensus events and tested whether the feature was statistically higher for non-consensus events using a one-sided Wilcoxon Rank-Sum test. Lastly, the threshold for separating false and true positive HFO detections was set to the average of (1) the upper quartile of the non-consensus events and (2) the lower quartile of the consensus events, which are quite close together. This threshold was selected as it labels about 75% of the consensus events as true positives and about 75% of the non-consensus events as false positives. We then called HFO detections with features above this value ‘putative true positive HFO detections’, and those below the value ‘putative false positive HFO detections’.

#### 2.5.2. Automated detection of putative true positive HFOs

The last validation steps were to assess whether putative true positive HFOs are less diffuse in general and have increased association with the SOZ and RV. Since the feature selection was accomplished agnostic to any HFO markings and the threshold was determined from human scored events using data from Cohort 2, the automated detections on Cohort 1 data were true out-of-sample test data for this validation step.

To quantify association of HFO rates with SOZ and RV, we employed the asymmetry measurement (Gliske *et al* 2018, Gliske *et al* 2016a, Ren *et al* 2019). This measure was applied per subject and was computed separately for the SOZ and RV. To determine the asymmetry, we first average HFO rate over all channels within the SOZ (or RV), denoted *r*_in_, and over all channels not in the SOZ (or RV), denoted *r*_out_. The asymmetry with respect to the SOZ (or RV) was then computed as A = (*r*_in_−*r*_out_)/(*r*_in_ + *r*_out_). This asymmetry measurement is bounded between −1 and 1 since the rates are non-negative, and higher values of the asymmetry indicate that the HFO rate is more localized within the SOZ (or RV). Statistical significance of the increase in asymmetry values was assessed using a one-sided Wilcoxon Sign Rank test.

We additionally recognize that SOZ and RV are not perfect gold standards of the epileptogenic zone. In other words, the epileptogenic zone is a theoretical concept (Jehi 2018) and standard clinical practice has no perfect way to directly identify its location. While SOZ and RV are the best standards available, they cannot be assumed to be 100% representative of the location of tissue causing seizures. For focal epilepsy, removing false positive HFO detections should make the remaining HFO detections more focal to a small set of channels. However, it is possible that this subset of channels may not overlap with the SOZ and/or RV as they are not perfect gold standards. Thus, we additionally assessed whether the HFO detections became more focal in general without specifying a specific subset of channels. We accomplished this by computing the Shannon Entropy, which decreases as distributions become more focal. We computed the entropy by normalizing the rate per channel such that the sum of rates was unity, and then computing the total entropy as −1 times the sum of *p*_*i*_ log_2_ *p*_*i*_, where *p*_*i*_ is the normalized rate for channel *i* (sum *p*_*i*_ = 1). Recall that *p*_*i*_ ln *p*_*i*_ = 0 for any *p*_*i*_ = 0. To account for variability in the number of channels across patients, we report the average entropy per channel (total entropy divided by the number of channels), which we denoted the normalized entropy. Statistical significance of the decrease in median of normalized entropy was assessed using a one-sided Wilcoxon Sign Rank test, a paired statistical test. We also assessed the likelihood that the change in entropy was due to the reduction in statistics. Per subject, we selected at random a subset of HFO detections per subject, keeping the fraction of randomly selected events the same as the fraction of putative true positive HFOs, and computed the change in entropy. We then repeated the process 10 000 times to compute a true p-value of the change in entropy per subject.

### 2.6. Code implementation

Preprocessing (including surrogate data generation), automated detectors, and quantitative feature computation for Cohort 1 data were all implemented in the General Data Flow Package (GDFP), written in C++ (Gliske *et al* 2016a). The code for the Burg method of computing autoregression coefficients was based on Oliver *et al* (2014) but modified by the authors in order to incorporate it into the GDFP. The remainder of the analysis was scripted in Matlab 2019b (Natick, MA). Code for creating replicates and for computing and analyzing quantitative features is posted at [location to be inserted later in the review process], along with the computed quantitative features for all detections.

## 3. Results

### 3.1. Feature selection

Features were selected based on the similarity between the distribution for zero bias (random sample) events and the HFO detections in the surrogate background data. Two features met the AUROC criterion: the skewness and kurtosis of the curvature of the signal, with median values of 0.517 and 0.522, respectively. The other features had AUROC values ranging from 0.604 to 1.000. We thus selected skewness of the curvature, which we abbreviate as skewCurve, as it has the AUROC value closest to 0.5.

### 3.2. Feature verification

The distribution of the skewCurve feature for HFO detections in actual interictal, intracranial EEG was decomposed into multiple fuzzy clusters using a Gaussian Mixture Model; see example in figure 3. We then quantified the relationship between the skewCurve distribution for zero bias and HFOs in surrogate background data with the component closest to them using AUROC, repeating the process for each subject. The median AUROC value was 0.53 (0.51–0.58, 95% confidence level (CI), 0.60 maximum) for comparison with zero bias events and 0.52 (0.50–0.56 95% CI, 0.57 maximum) for comparison with HFO detections in the surrogate background data. Thus, in each subject, one component of the actual HFOs nearly identically matches the distributions based on background data.

### 3.3. Validation using human-scored HFO detections

**Figure 4.**
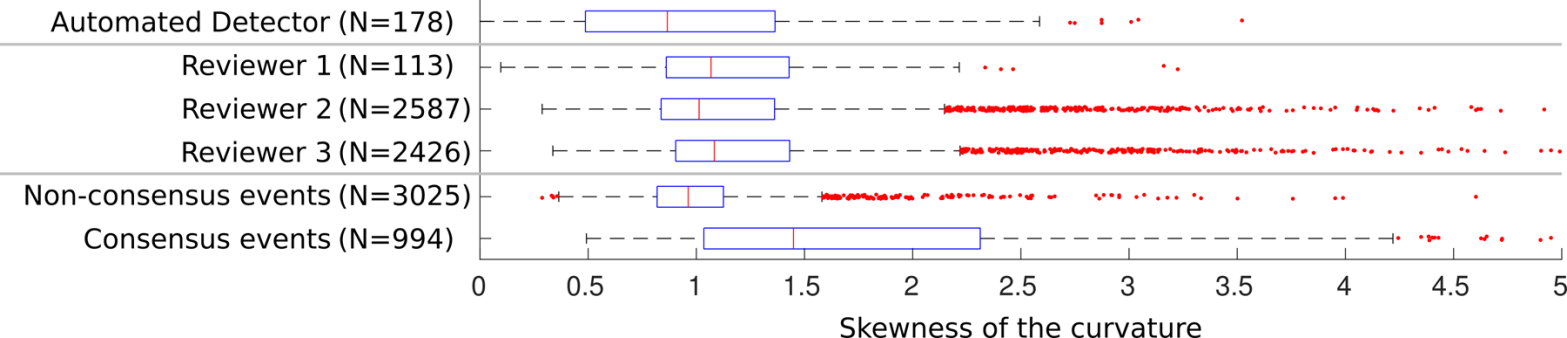
Box plot of skewCurve in Cohort 2 data. HFOs were manually scored in 20-minutes of 2 channels of intracranial, interictal EEG by three reviewers. The skewCurve feature was computed for these events and for automatically marked HFO detections in these patients. The number of events are indicated for each row of the box plot. Red line indicates the median, the blue box spans from the lower to the upper quartile, and the whiskers extend past the quartiles by 1.5 times the interquartile distance. Data beyond the whiskers are marked as red dots.

The skewCurve distribution for the Cohort 2 data is shown in figure 4. We next sought to validate the skewCurve feature by testing the hypothesis that low skewCurve values were associated with false positive HFO detections and high skewCurve values were associated with true positive HFO detections. While human reviewers marked less data with low skewCurve than did the automated algorithm, they still marked some: the median value of skewCurve was near 1.0 for all three reviewers (cf. figure 3). We also observed that the skewCurve distribution was relatively uniform for each reviewer (AUROC < 0.61 for each binary comparison), despite the wide range of number of HFO events marked (from *N* = 113 to *N* = 2587). However, events which were marked by more than one reviewer had much higher skewCurve values than events marked by only one reviewer: the AUROC value between consensus and non-consensus events was 0.79, and the difference in medians was highly significant (p < 10^−160^, one-sided Wilcoxon Rank Sum). The data thus strongly support the hypothesis, validating that the skewCurve feature differentiates between false positive and true positive detections.

We observed that the upper quartile mark for non-consensus events (figure 4) is near the lower quartile mark for consensus events. We averaged these to quantities to obtain a threshold of skewCurve = 1.08 to separate false and true positive HFO detections. This threshold was set based on Cohort 2 data using manually scored HFOs, with the automated detections in Cohort 1 data being independent validation data for assessing the impact of redacting data below this threshold. We also observe that about half of the events marked by any one reviewer would be classified as false positives by this threshold, as well as over half of the automated HFO detections in this subset of Cohort 2 data.

### 3.4. Automated HFO detections

**Figure 5.**
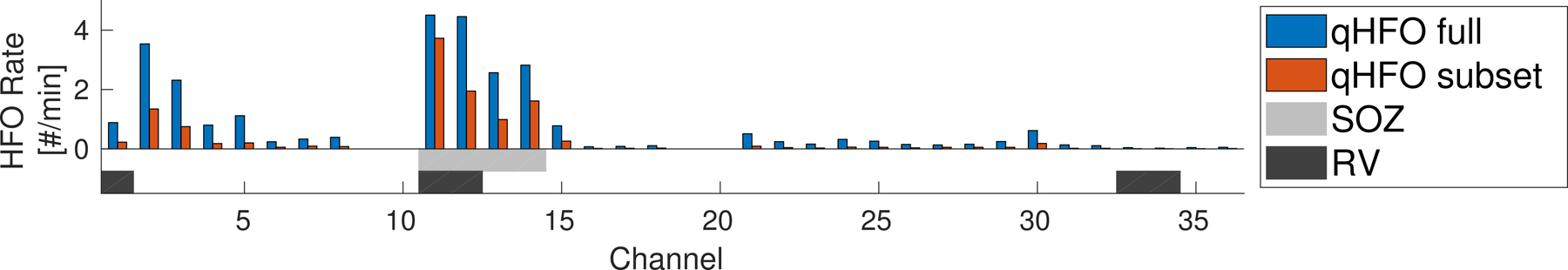
Example HFO rates. The HFO rate per minute is shown for both the full set of qHFO detections (blue) and the putative true positive subset (rust color). Channels within the SOZ and/or RV are indicated by colored boxes underneath the *x*-axis. The putative true positive qHFOs are focused on a few channels, whereas the full set of qHFOs are more diffuse (e.g. channels 20–30).

The effect of selecting the putative true positive subset of qHFOs (i.e. skewCurve > 1.08) is shown for an example patient in figure 5. The qHFO detections were somewhat diffuse, with many channels having an observable HFO rate and six channels being quite high (>2 HFO detections per minute). The subset of putative true positive HFOs, however, had a negligible rate on most channels and only a few channels with substantial HFO rates.

**Figure 6.**
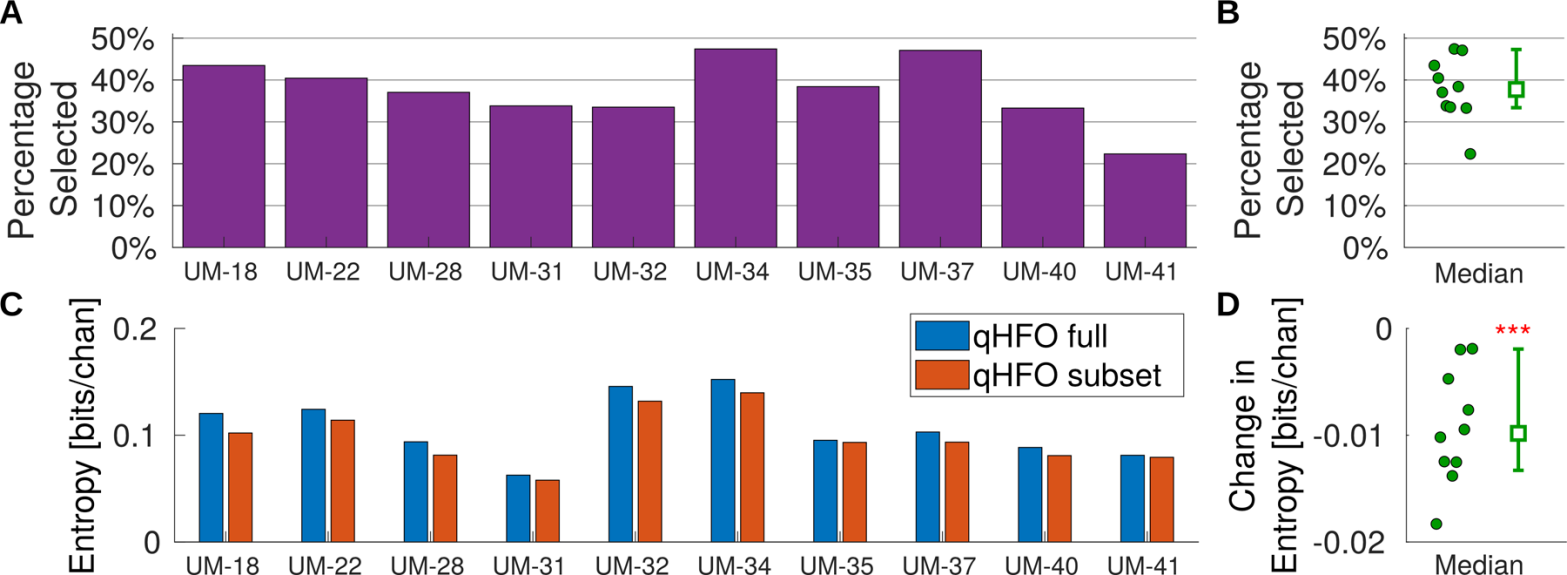
Percentage of events remaining and entropy of HFO rates per channel. The fraction of automated HFO detections in the putative true positive subset are reported for each subject (A), along with the median and its 95% CI (B, open squares with an uncertainty bar). The value per patient (data in A) are also shown in (B) as filled circles for reference. Additionally, the normalized entropy (total entropy/number of channels) is shown for both the full set of qHFO detections and the subset of putative true positive events (C). The change in normalized entropy is also provided (D) per patient (filled circles) along with the median and 95% CI, drawn with an open square with uncertainty bar. Three asterisks indicate that the median change is different than zero with very high significance (p = 0.001, one-sided Wilcoxon Rank Sum).

We next assessed the percentage of HFO detections remaining after redacting putative false positive HFO detections. The percentage per patient is shown in figures 6(A) and (B), and the median percentage was 38% (95% CI 33% to 47%).

We next quantified whether the putative true positive HFOs were less diffusely distributed over channels; see figures 6(C) and (D). A decrease in entropy indicates a more focal, less diffuse distribution, which was the case for all ten subjects. The median of the normalized entropy decreased 0.010 with 95% CI of 0.002 to 0.013. This median change is statistically different than zero (p = 0.001, one-sided Wilcoxon Sign Rank) and not due to the reduction in statistics (all subjects p < 0.0001, random subsets). See figure 6(A) for the specific reduction in statistics per subject. Thus, the data support that the putative true positive subset of HFOs were more focused on a subset of channels.

### 3.5. Comparison with SOZ and RV

**Figure 7.**
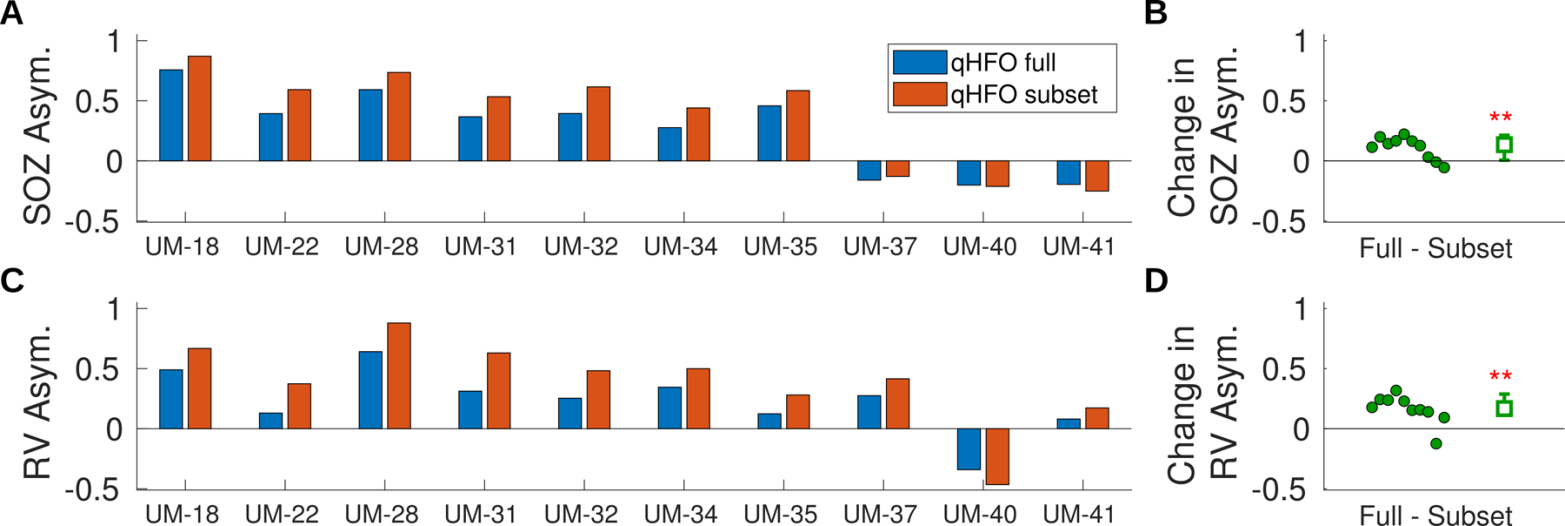
HFO Rate Asymmetries. The asymmetries are computed with respect to the SOZ (A) and (B) and RV (C) and (D). The asymmetries are shown for both the full set of qHFO detections and the subset of putative true positive events per patient (A) and (C). Additionally, the change in asymmetry is shown for each patient (B) and (D) as a filled circle. The median change with 95% confidence-level (CI) is shown as an open square with uncertainty bars. Two astePatient demographic data.risks indicate that the median is different than zero with high significance (p = 0.007 (C), p = 0.003 (D), one-sided Wilcoxon Sign Rank).

The most important validation was the clinical validation: assessing whether the putative true positive HFOs have increased association with the epileptogenic zone. We quantified this association by computing the asymmetry of the HFO rate with respect to the SOZ and RV; see figure 7. When comparing with SOZ, eight of ten subjects had a higher asymmetry for the subset of putative true positive HFOs, indicating an increased specificity towards SOZ. The two patients that did not increase in asymmetry with respect to the SOZ still had the HFOs become more focal to known diseased tissue, as detailed in the following paragraph. The median of the asymmetry with respect to SOZ increased by 0.14 (95% CI 0.01–0.21), and we rejected the null hypothesis of no increase with strong statistical significance (p = 0.007, Wilcoxon Rank Sum).

The results for comparing with RV were similar: nine of ten subjects had an increase in asymmetry. The one subject that did not increase in asymmetry is the one subject with negative asymmetry rate, UM-40, described below. The median asymmetry with respect to RV increased 0.17 (95% CI 0.11–0.29), rejecting the null hypothesis of no increase (p = 0.003, one-sided Wilcoxon Sign Rank). The data thus support that the subset of putative true positive HFOs have an increased association with the epileptogenic zone.

The two patients whose asymmetry did not increase both had complicated surgeries due to radiographic lesions, and the HFOs still had clinical value. The patient that had a slight decrease in both asymmetries (UM-40) had an ECoG grid over a large parietal dysplasia/infarct and a hippocampal depth electrode. The full set of qHFOs were somewhat focal to the hippocampus, and the effect of redacting the putative false positive HFOs in this subject was to cause the HFOs to be more focal to the hippocampus. The hippocampus was not marked as part of the primary SOZ, but rather was noted in the clinical report as being quickly recruited into seizures and also had many interictal spikes. It was not resected because the patient was already getting a large parietal lesionectomy, but it was clearly active in the seizure network. Thus, the decrease in asymmetry was due to the hippocampus remaining; this does not represent a failure of HFOs or the method of identifying putative false positive detections, but rather that the patient was fortunate that the parietal resection disrupted the seizures, even though there was clinical suspicion that the hippocampus was also involved. The other patient with a slight decrease (UM-41) had a schizencephaly over several electrodes, but only one electrode was resected. The full set of HFOs was localized to the same stereo-EEG electrode as the SOZ, all of which was within the radiographic lesion but in more superficial contacts than the described SOZ. However, all of the contacts on that electrode were included in the resection. Redacting putative false positive detections caused the HFO rates to be even more focal to these superficial channels. Again, the HFOs became more focal to a region known to be pathological, even though the regions were not within the clinically-marked SOZ.

## 4. Discussion

The goal of this paper was to identify and validate quantitative HFO features to identify and redact false positive detections in order to improve the association of HFOs with epileptic tissue. The goal was accomplished, and the identified feature was skewCurve. This quantitative feature is equally applicable whether the HFO was identified by a human or the analyzed automated algorithm. This feature was selected by analyzing background data, specifically randomly selected samples of the background and running the HFO detector on surrogate background data generated based on the actual background data. HFO detections in the actual EEG data were found to have a natural cluster that directly matches the distributions from the background data. Additionally, this feature’s association with true and false positive detections was validated by analysis of human marked HFOs. The human data analysis in Cohort 2 subjects (not ILAE Class 1) also allowed an independent sample to train the threshold for separating putative true and false positive HFOs. Applying this threshold to Cohort 1 subjects (all ILAE Class I), the putative true positive HFOs were less diffusely distributed over channels and had increased association with pathological tissue (SOZ, RV, MRI lesion or region with many interictal spikes and fast seizure spread).

Our analysis has a few minor limitations. One of these is that we only tested one automated HFO detector. However, we note that the procedure included seeking a close match with the distribution of randomly selected samples of the EEG background (the zero bias events), which is completely agnostic to the specific HFO detector. Thus, we anticipate that the results will generalize across HFO detectors. Furthermore, the threshold for separating true and false positive HFOs was based on human scored HFOs, again agnostic to the automated HFO detector. Therefore, the results are again expected to apply to any automated HFO detector. We also observed that among the human reviewers, one reviewer marked 113 events, whereas the other reviewers marked over 2000. We thus see that among expert human reviewers of HFOs, there is great variety in sensitivity. However, the distribution of skewCurve for each person was very similar. These data further support that the results will generalize to other methods of detecting HFOs, as it generalizes well across several very different human reviewers. Another minor limitation is that we only tested 15 quantitative HFO features. Future work may yet discover other new, untested features which may prove even more useful in identifying false positive detections. Additionally, a more nuanced approach for selecting the length of the zero bias events could possibly lead to additional useful features among the given set. However, such possibilities do not weaken the conclusions of the present analysis. Lastly, another limitation is that data were only analyzed from one acquisition machine at one center. While we expect that acquisition equipment may lead to a slightly different threshold, we expect the general results to still hold. Furthermore, one can easily verify whether the threshold is appropriate for data acquired on other machines at another center by computing the skewCurve on a random selection of 20 msec segments of bandpass filtered, background EEG data and observing if the distribution is different than that observed in our data.

Given the many constraints in our feature selection procedure, we expected few, if any features, would be selected. HFOs are distinct from background data, and thus it is expected that most features would be very different between HFOs and background. However, the distribution of skewCurve was similar between the HFOs and background events. This may be due to several properties of the feature. First, it is agnostic to both the magnitude and DC offset of the data. Second, it is also nearly agnostic to the time-scale, with correction terms proportional to the square of max frequency (500 Hz) over sampling rate (4 kHz). This means that two signals which are the same shape, with one being just a zoomed-in version of the other, would have the same skewCurve. It also means that skewCurve is practically invariant to differences in sampling rate. These properties allow skewCurve to assess the general shape of an HFO without being dependent upon amplitude or frequency scale.

One of the important, novel aspects of this manuscript is how background data is used to determine characteristics of false positive detections. In our analysis, we created alternate background data (the surrogate data) on which to apply our actual HFO detector and we also used an alternate detector (the zero bias detector) on the actual background data. This use of background and of replacing portions of the regular analysis stream with alternate methods allowed us to assess additional information about the detection process, specifically characteristics of false positive detections.

We note that there are a variety of different types of false positive detections. Our experience has been that each type of false positive event requires a unique approach. Previous publications addressed visually-observable false positive events, such as those due to sharp transients and filtering effects (Gliske *et al* 2016a), and muscle artifacts (Ren *et al* 2019). The analysis of this paper focused on a type of false positive detection that is not readily observable by eye: random fluctuations of the background. These methods of this paper generalize to other detectors and data where random fluctuations of the background data could cause false positive results.

Our use of background data is also unique from that done in other HFO research. HFOs are defined as discrete events, and thus HFOs are different from the background in multiple ways. Various HFO detectors utilize background data to identify anomalies as putative HFOs. In fact, the heart of our qHFO algorithm is a method based on Staba *et al* (2002), which involves setting thresholds based on the background. Background data is also used in the qHFO detector to identify and redact HFO detections due to fast transients and non-focal HFOs. Other detectors also analyze the background to improve HFO detection, such as to identify periods with minimal oscillatory activity to estimate baseline activity (Zelmann *et al* 2010) or to clarify the context of the HFO and identify false positive detections from specific types of noise (Liu *et al* 2016). Analysis of the background is also involved in studies testing the utility of restricting to only HFO occurring on a spike (Wang *et al* 2013) or repetitive pattern (Liu *et al* 2018). An alternate approach is to use analysis of background as an alternative to using an HFO detector for assessing HFO information (thus circumventing the challenge of false positive detections), and instead compute features of the combined background plus HFO signal (Akiyama *et al* 2012, Geertsema *et al* 2015, Mooij *et al* 2020, Xiang *et al* 2020). Unique to the analysis of this manuscript is that we directly assessed the background properties to learn characteristics of false positive HFOs due to neural sources, rather than using it solely for establishing a baseline, for removing false positives due to artifacts (non-neural sources) or as an alternative to HFO detection.

Additionally, few analyses have investigated quantitative features of HFOs to improve specificity to the epileptogenic zone. Some include difficult to reproduce training data (e.g. Matsumoto *et al* (2013)) and/or complex machine learning algorithms (e.g. Blanco *et al* (2011) and Liu *et al* (2018)). Additionally, Liu *et al* (2018) analyzed quantitative features at the time of HFOs, but used a wide frequency range. Those results are thus likely dominated by lower frequencies (below those of HFOs) which have higher power, and thus are not actually quantitative features of the HFO. Another study limited analysis to a set of features designed to recapitulate a visual categorization of HFOs (Burnos *et al* 2016). Instead, the results of this paper include a simple threshold on a single, easily-computed quantitative feature. Identification of putative true positive HFOs using our method can thus be easily incorporated into any research or clinical protocols that already include digital processing of EEG and identification of HFOs. These results do not remove the possibility that physiological HFOs may still be present, but removal of false positive detections may help facilitate future research to distinguish between physiological and pathological HFOs. Our results thus move the field closer towards the development of generalizable rules for identifying the most relevant HFO detections.

## Figures and Tables

**Table 1.** Patient demographic data.

Patient	Cohort	Age	Sex	Resection	Outcome	Electrode Location (and Type)	Pathology
UM-18	1	41	M	L Frontal Cingulate	I	L and R frontal (depths)	CD
UM-19	2	59	F	R ATL	II	R temporal, parietal, occipital (grids, strips)	Mild gliosis, multifocal (right parietal focus not resected)
UM-20	2	45	F	R ATL	II	R parietal, occipital, mesial temporal, insular (depths)	MTS
UM-21	2	30	M	R ATL	II	R temporal, insular (depths)	Gliosis, polymicrogyria, PVNH
UM-22	1	40	M	L ATL	I	L temporal (depths)	Mild CD and MTS
UM-23	2	29	M	none	n.a.	L temporal, parietal (depths, grids, strips)	Mutlifocal: parietal and hippocampal
UM-24	2	31	M	none	n.a.	L temporal, parietal, occipital (grids), L&R mesial temporal (depths)	
UM-25	2	17	F	L Temporal	II	L&R mesial temporal (depths)	Gliosis
UM-28	1	14	F	R ATL	I	R temporal, parietal (grids, depth)	Low grade glioma
UM-31	1	13	M	L ATL	I	L temporal, parietal (grids, strips)	Gliosis within resection, NF1 outside of resection
UM-32	1	41	F	R Frontal	I	R frontal, insular (depths)	CD
UM-34	1	33	F	R Frontal	I	L frontal (depths)	Gliosis
UM-35	1	50	F	L AH	I	L frontal, temporal (grids, strips)	Gliosis
UM-37	1	14	M	L Frontal	I	L frontal, temporal (grids) frontal, insular (depths)	DNET
UM-40	1	14	F	L Parietal	I	L parietal, temporal (grid), mesial temporal (depth)	CD and gliosis
UM-41	1	32	F	R Frontal	I	L frontal, cingulate	CD

Abbreviations: ATL anterior temporal lobectomy; AH amygdalohippocampectomy; CD cortical dysplasia; DNET dysembryplastic neuroepithelial tumor; MTS mesial temporal sclerosis; PVNH periventricular heterotopia; n.a., not applicable; NF1 neurofibromatosis type 1.

## Abbreviations

CI: confidence interval
HFO: high frequency oscillation
qHFO: a HFO event detected using the quality-HFO detector
RV: resected volume
SOZ: seizure onset zone
SkewCurve: the skewness of the curvature feature
